# Evolution of increased positive charge on the SARS-CoV-2 spike protein may be adaptation to human transmission

**DOI:** 10.1016/j.isci.2023.106230

**Published:** 2023-02-18

**Authors:** Matthew Cotten, My V.T. Phan

**Affiliations:** 1 Medical Research Council–University of Glasgow Centre for Virus Research, 464 Bearsden Road, Glasgow G61 1QH, Scotland, UK; 2 UK Medical Research Council–Uganda Virus Research Institute and London School of Hygiene and Tropical Medicine Uganda Research Unit, Plot 51- 59 Nakiwogo Road, P.O Box 49, Entebbe, Uganda, UK

**Keywords:** Evolutionary biology, Virology

## Abstract

The severe acute respiratory syndrome coronavirus 2 (SARS-CoV-2) continues to evolve and infect individuals. The exterior surface of the SARS-CoV-2 virion is dominated by the spike protein, and the current work examined spike protein biochemical features that have changed during the 3 years in which SARS-CoV-2 has infected humans. Our analysis identified a striking change in spike protein charge, from −8.3 in the original Lineage A and B viruses to −1.26 in most of the current Omicron viruses. We conclude that in addition to immune selection pressure, the evolution of SARS-CoV-2 has also altered viral spike protein biochemical properties, which may influence virion survival and promote transmission. Future vaccine and therapeutic development should also exploit and target these biochemical properties.

## Highlights

• Biochemical features of the SARS-CoV-2 spike protein have changed during the pandemic
• Spike charge has steadily increased from −8.3 to −1.26 in Omicron lineages
• Multiple spike functions may be altered: epitopes, receptor binding, furin cleavage
• Other coronaviruses moving from animals to humans show similar spike charge increases

## Graphical abstract

**Figure:**
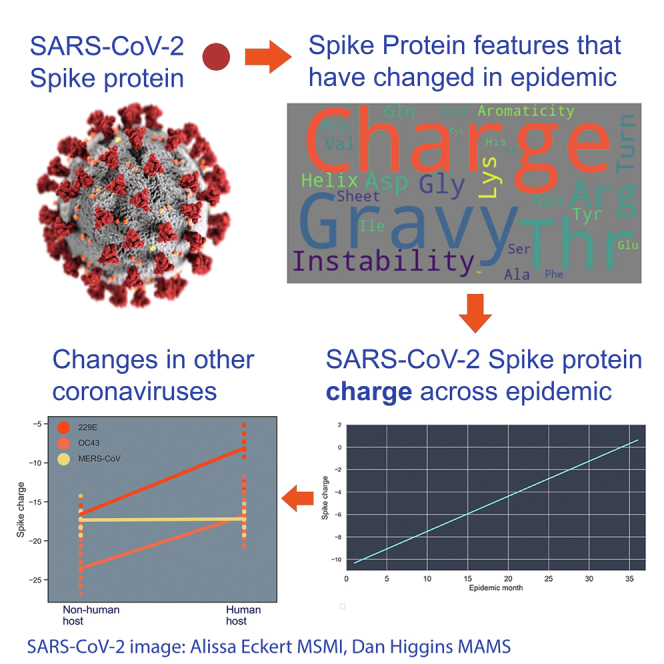

## Introduction

The severe acute respiratory syndrome coronavirus 2 (SARS-CoV-2), the causative agent of the coronavirus disease 2019 (COVID-19) epidemic, continues to evolve and infect individuals. Similar to other viruses, the SARS-CoV-2 virion biochemical properties play an important role in controlling virus transmission. After replication in an infected individual and release from an infected cell, onward transmission requires survival of the virion to reach susceptible cells in a new host individual initiating the next round of infection. The physical properties of the surface proteins of the virus such as charge, size, hydrophobicity, and folding may influence movement of the virion through the environment, promoting or limiting binding of the virion to the external surfaces. Once reaching a susceptible individual, physical properties of the virion may influence movement within the human airway and determine the ability of an infecting virion to reach target cells to bind, enter, and replicate.^1^ The exterior surface of the SARS-CoV-2 virion is dominated by the spike protein, and the current work examines simple spike protein features that have changed during the nearly 3 years of the SARS-CoV-2 pandemic. In addition to selective pressure to avoid immune recognition of viral proteins, we hypothesize that SARS-CoV-2 emerged from an animal reservoir capable of human infection and transmission but in a sub-optimum state. Additionally, there is a second level of selective pressure to adjust to the physical transmission between humans. Evidence for this adaptation can be found in changes in the SARS-CoV-2 spike protein over recent evolution. With over 14 million SARS-CoV-2 genomic sequences generated globally from across the pandemic, many of these sequences have intact spike gene sequences that can be used to monitor change across the nearly 3 years of human host evolution of this virus.

Much of the observed spike protein substitutions may be in response to the developing immune response to this new pathogen, which is reflected in substitutions occurring in the immune-exposed S1 domain of the spike protein, and there is ample evidence that many of these spike protein changes allow escape from host immunity.^2^^,^^3^^,^^4^^,^^5^^,^^6^^,^^7^^,^^8^ There may also be evolutionary selection for protein changes that improve host interactions apart from immune evasion. These include altering spike-receptor binding kinetics, protease cleavage events, tertiary structure (S1/S2 interactions after cleavage), or the physical properties of the virion (charge, hydrophobicity, and protein folding or secondary structure) in ways that might improve transmission. To explore the role of the biochemical features of the spike protein in human transmission, we monitored changes in spike biochemical features over the two years in which SARS-CoV-2 has been evolving in humans and report an increase in spike protein positive charge especially among the virus lineages that were highly prevalent.

## Results

### Identification of spike protein charge association with SARS-CoV-2 lineage

The SARS-CoV-2 spike protein physical features were calculated from spike protein sequences from across 3 years of the COVID-19 epidemic. Features that could be quantitated from the protein sequence were used (see STAR Methods), including charge at pH 7.4, Kyle and Doolittle GRAVY score^9^ (which is a measure of hydrophobicity), an instability index derived from the dipeptide content,^10^ properties influencing protein folding (percent helix, fold, or sheet as predicted from amino acid [AA] content), individual AA total fraction, and di-amino acid total fraction.

**Figure 1:**
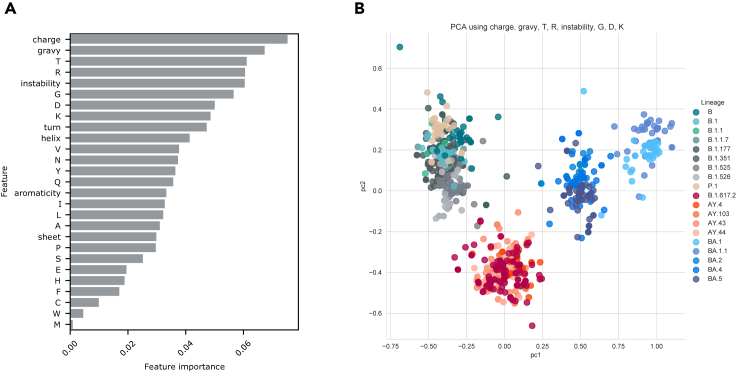
Identification of spike protein charge association with SARS-CoV-2 lineage (A) A set of 300 spike sequences extracted from the first 300 SARS-CoV-2 genomes per lineage (by date of collection) was analyzed, and features for each sequence were collected (see STAR Methods). SKLearn feature selection^41^ was used to identify features that most accurately identified the sequence lineage. The importance of features was ranked in order. (B) The top 8 features (charge, gravy, fraction T, fraction R, instability, fraction G, fraction D, and fraction K) were further used in a principal component analysis to cluster the same set of SARS-CoV-2 spike sequences. Each node represents a single spike sequence, and nodes were colored by Pangolin lineage assigned to the genome from which the spike sequence was obtained. Lineage coloring is explained in the right side of the panel. The proportion of variance explained by the first principal component was 64%, and for the first and second principal components, the proportion of variance explained was 84%.

A dominant pattern of SARS-CoV-2 evolution during the three years of human adaptation has been the regular appearance and the subsequent regional and then global dominance of lineages. These lineages typically encode a small set of AA changes from earlier lineages, many of which are likely to provide temporary or long-term advantages for the viral lineage. An analysis was performed to identify spike physical features most strongly linked with SARS-CoV-2 lineages (Figure 1). The first 300 reported genomes from each major lineage were collected, spike protein sequences were extracted, and the physical features of each protein were collected into a matrix. The top features distinguishing SARS-CoV-2 lineages were identified, with charge as the most important feature (Figure 1A). A principal component analysis using the top 8 features (charge, gravy, fraction T, fraction R, instability, fraction G, fraction D, and fraction K) provided clustering of spike sequences by lineage (Figure 1B). An iterative method of determining the accuracy of the classification by the number of features was used to select the most important features, and this showed that 8 features provided nearly the maximum accuracy for classification with only incremental improvements beyond these 8 features (Figure S1). These results support the idea that spike protein charge (among other features) is an important determinant of the lineages that have evolved during the first three years of the COVID-19 epidemic.

### Total SARS-CoV-2 spike charge per epidemic month

**Figure 2:**
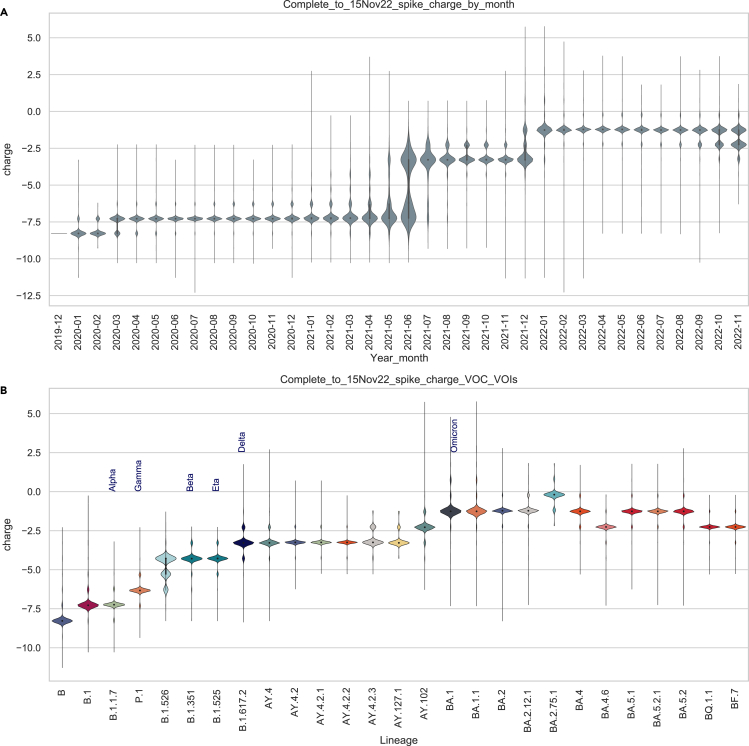
Total SARS-CoV-2 spike charge by epidemic month and by lineage (A) Total SARS-CoV-2 spike charge per epidemic month. All available SARS-CoV-2 genomes up to November 15, 2022, were retrieved from GISAID,^42^ and the spike protein sequence was extracted (if intact). Total charge at pH 7.4 was calculated, and values were plotted using a violin plot by month of sample collection. For each epidemic month, the violin plot depicts the distributions of calculated spike charge for all available SARS-CoV-2 genomes. (B) Spike charge in major SARS-CoV-2 lineages. For each lineage, all available spike sequences were collected (up to November 15, 2022). Total charge at pH 7.4 was calculated, and violin plots were prepared to show the charge distribution by lineage. Lineages (indicated at bottom of chart) were ordered by their appearance in the epidemic. The first lineages of the main variants of concern and variants of interest are also labeled in the figure.

Changes in charge of spike protein across the epidemic were investigated. Plotting total spike charge for all genomes per month of the epidemic showed a clear pattern of increase in charge over three years of evolution (Figure 2, panel A). Median spike charge was −8.3 in the original SARS-CoV-2 viruses reported in late 2019 to early 2020, but by March 2020, an increase in positive charge to −7.28 was observed. Subsequently, an additional increase in positive charge occurred in mid-2021 to −3.28, and most recently, a charge increase occurred in late 2020/early 2021 to −1.26.

### Spike charge in major SARS-CoV-2 lineages

These spike protein charge increases can be attributed to the major successful lineages reported over time (Figure 2B). The B.1, B.1.1, and B.1.1.7 (Alpha) lineages that dominated the first year of the epidemic encoded spike proteins with charges between −8 and −6 while the B.1.351 (Beta) and B.1.525 (Eta) lineages showed a further increase in charge to around −4.5. The B.1.617.2 (Delta) lineage and sub-lineages (AY.x) displayed further increases in charge. Most recently, the Omicron variants (including the most abundant omicron lineages BA.1, BA.1.1, BA.2, BA.2.12.1, BA.2.75.1, BA.4, BA.4.6, BA.5. BA.5.1, BA.5.2.1, BA.5.2, BQ.1.1, and BF.7) show further spike charge increases over the Delta lineages, with the majority of Omicron-encoded spike proteins showing charge at −1.26 (Figure 2, panel B). Further variations in Omicron sub-lineages are discussed below.

### Location of charge changes

Some indication of functional consequences of the observed changes in spike charge can be obtained from the location on the charged AA substitutions in the spike protein. Sets of spike sequences (extracted from the first 300 reported genomes per select lineage) were processed to illustrate the changes to a more negative charge (blue) or more positive charge (orange/red) in the protein relative to the initial Lineage B genome sequences (Figure S2). The initial change in charge was a substitution of an aspartic acid residue (D, with a calculated charge of −1) by glycine (G, neutral). In some early lineages (e.g., A.23.1), proline (P) at position 681 was substituted with the positively charged arginine (R), or Q680 was substituted with a partially charged histidine H residue. The P681R positive substitution promotes furin cleavage and activation of the spike protein for cell fusion.^11^^,^^12^ The Delta lineage spike proteins encoded additional positive charge in the ACE2 binding region, as well as in the far amino terminal region and near the heptad repeat (HR1), which may also enhance membrane fusion activity. More recently, a number of positive substitutions have occurred in the Omicron lineage virus spike proteins, with predominance of positively charged changes in the receptor binding domain (Figure S2), suggesting a role of increased charge in spike-receptor interactions.

### pH dependence

The spike charge calculations were performed using a pH value of 7.4; however, the virus may encounter other pH values from 7.5 to 5.7 during human infection (see Table S3).^13^^,^^14^ Accordingly, we monitored the pattern of total spike charge across the epidemic for pH 7.4, 6.6, 6.0, and 5.7 and show that the pattern of increase in positive charge remains the same, only the curves are shifted to more positive charge as the pH is decreased (Figure S4). The magnitude of the charge difference between the original lineage B SARS-CoV-2 and the later lineages remains the same, and our conclusion that SARS-CoV-2 has evolved to increased positive charge on the spike protein is independent of the pH used for the calculation.

### Recent changes in spike protein charge

**Figure 3:**
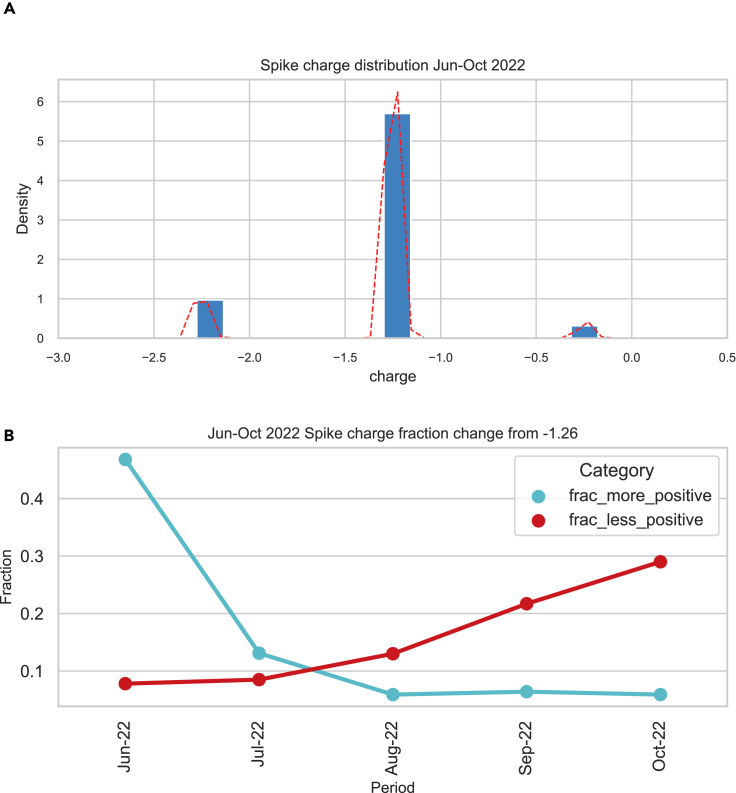
Recent changes in spike protein charge (A) All available spike proteins from genomes with sample collection dates of June to October 2022 were analyzed for total spike charge. A histogram of the calculate total spike charges for the entire set is shown here with the kernel density estimation (KDE) line in red. A major peak at −1.26 is observed, with small outlier peaks of genomes with more negative and more positive spike proteins. (B) For each month (over the period June 1 to October 31, 2022), the fraction of reported genomes for that month with charge greater than or less than the majority value of −1.26 was calculated.

It is probable that the spike protein has an upper limit to the AA charge that it can allow for proper folding, assembly, and function. This upper charge value will be determined by the acquisition of optimum virus replication and transmission properties in balance with immune selection. After the regular increase of spike protein charge observed up to the appearance of the Omicron lineages, an indication of a stasis in positively charged AA accumulation is now displayed by SARS-CoV-2 Omicron lineages. The majority of Omicron sub-lineages remain at a spike charge of −1.26 (Figure 3A) although a few specific Omicron sub-lineages show changes toward a more positive or negative charge (e.g., BA.2.75.1 more positive, BA.4.6 more negative, as illustrated in Figure 2B), with the additional changes often associated with immune selection. To monitor the current trends of spike protein changes, we calculated the fraction of reported genomes with spike charge greater than or less than the Omicron mean charge of −1.26 and documented how these fractions had changed over the last 4 months of the pandemic (Figure 3B). The majority of encoded spike proteins are almost exclusively from Omicron lineage viruses and show a charge of −1.26. However, a small fraction of genomes encode spike proteins with slightly more or less charge (Figure 3B), with the greater trend (almost 30% of all reported genomes in October 2022) showing more negative charge (Figure 3B).

### Spike charges from select groups of coronaviruses that have moved into humans

**Figure 4:**
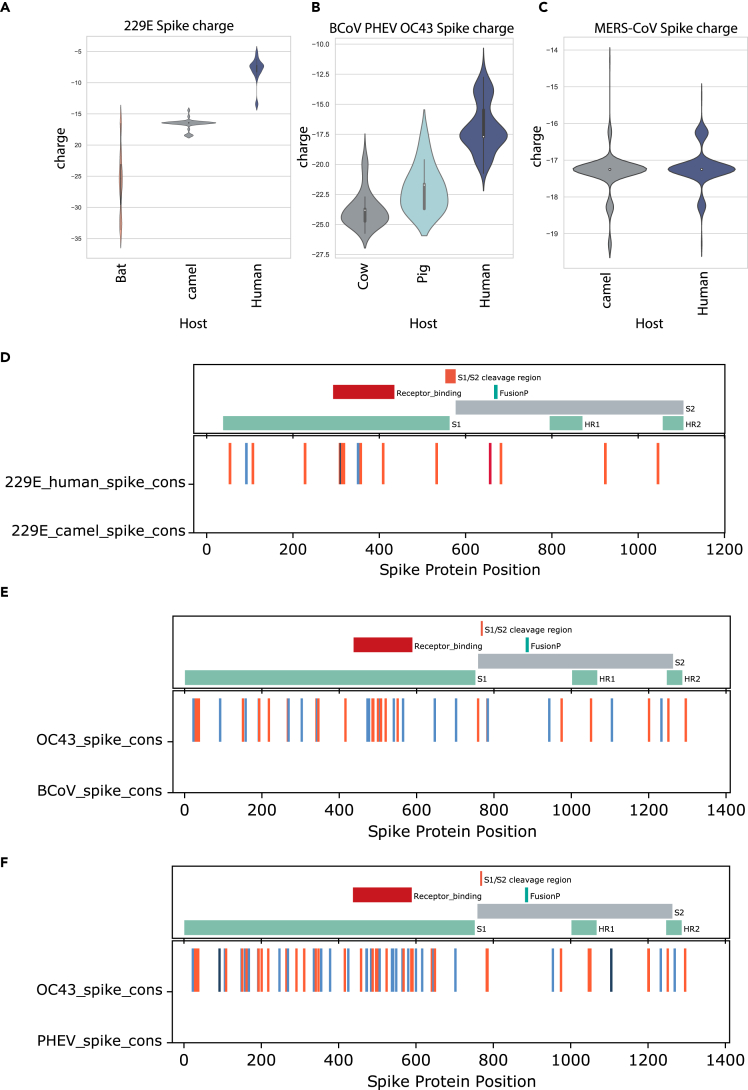
Spike charges from select groups of coronaviruses that have moved into humans (A–C) All available full genomes for the indicated coronaviruses were retrieved from GenBank; the spike coding region was identified and translated into protein; and total charge at ph 7.4 was calculated. Violin plots indicate the charges of each collection of spike proteins; median values are indicated by the open square. (A) Coronavirus 229E from bat, camel, or human infections; (B) BCoV (from bovine infections), PHEV (from porcine infections), and OC43 (from human infection); (C) MERS-CoV from camel or human infection. (D–F) Consensus spike protein sequences were generated from the indicated virus groups, and charged amino acid (AA) changes were determined. Charge changes were colored dark blue (change from positively to negatively charged AA, blue change from neutral to negatively charged AA), orange (change from neutral to positively charged AA), and red (change from negative to positively charged AA). (D) 229E Spike from human infections compared to 229E spike from camel infections; (E) human OC43 spike compared to BCoV spike; (F) human OC43 spike compared to PHEV spike. Key spike protein features of each group’s spike protein are shown in the upper portion of each panel.

Lastly, we investigated if a similar pattern of spike charge evolution could be observed in other coronaviruses that have made a transition to human transmission. In recent history, several coronaviruses (in addition to SARS-CoV-2) have been observed to jump hosts. For example, coronavirus 229E is commonly detected in humans, and very close coronaviruses have been identified in bats^15^^,^^16^ and camels,^17^^,^^18^ suggesting movement of the virus between hosts. All available coronavirus 229E full genomes sequences were retrieved from GenBank, and the spike coding region was extracted from the genomes, translated, and total charge was calculated. A difference from −26 to −8, or almost 18 charge units, is seen comparing 229E-like viruses from bats to 229E from humans (Figure 4A), and almost a 9-charge-unit difference was observed in spike median charge comparing 229E viruses from camel vs. human infections (Figure 4A).

Infection with coronavirus OC43 is common in humans, and closely related viruses are found in cattle (bovine coronavirus [BCoV])^19^ and pigs (porcine hemagglutinating encephalomyelitis virus [PHEV]). ^19^^,^^20^ Comparing the three OC43-type virus groups, the human virus OC43 has an increased charge of ca. 5 units compared to PHEV and ca.7 units compared to BCoV (Figure 4B).

The commonly known host for the Middle Eastern Respiratory Syndrome coronavirus (MERS-CoV) is dromedary camels; however, zoonosis and serious human infections occur frequently^21^^,^^22^^,^^23^^,^^24^^,^^25^ as reviewed by Peiris and Perlman.^26^ From 698 full MERS-CoV genomes available in GenBank, there was no strong difference in the encoded spike charge of virus sequences derived from human vs. camel infections (Figure 4C).

Considering the location of the charge differences in the spike proteins, for coronavirus 229E, the charge increases occurred throughout the protein although there is a slightly higher number of positive changes in the receptor-binding region of the human-infection-derived viruses (Figure 4D). For OC43, the porcine and human viruses also show increases in positive charge throughout the spike protein, and the porcine PHEV also showed a slight enrichment in positive charge in the receptor-binding region (Figure 4E).

## Discussion

After three years of the COVID-19 pandemic and with the availability of >14 million SARS-CoV-2 genome sequences, a trend of SARS-CoV-2 spike protein charge can be observed, with successive lineages showing an increase in positive charge over earlier lineages. Over the course of the pandemic, the SARS-CoV-2 spike protein has evolved from a protein with a total charge of −8.28 in the original Lineage A and B viruses to a protein with a total charge of −1.26 in the majority of the currently circulating Omicon lineage viruses. This pattern has been noted previously.^27^^,^^28^ We expand on these observations; document lineage patterns and sites of change in the spike protein; and explore similar phenomena of evolution to more positive charge in two other coronaviruses (OC43 and 229E) that have moved between animals and humans.

This study does not identify a mechanistic basis for the increased spike charge although there are several possible transmission steps that might be promoted by increasing charge. Exposed, positively charged spike AAs should promote interactions with negatively charged cellular structures. Interactions with negatively charged heparin have been reported with SARS-CoV-2 spike,^29^ and negatively charged sialylated glycans are reported to promote entry of SARS-CoV-2.^30^ The upper respiratory tract is coated and protected by mucins, frequently modified with sialic acid or phosphorylated, high-mannose N-glycans^31^ which present a negatively charged matrix that could either promote or protect against viral transmission. The SARS-CoV-2, OC43, and BCoV virions display binding to negatively charged carbohydrate structures found in the airway,^31^ and the ionic environment of the human upper respiratory tract may favor binding and transmission of viruses with increased positive charge. Perhaps it is not surprising that both OC43 and 229E coronaviruses exhibited increases in spike positive charge after moving from animal hosts (cows, pigs, and camels) to human hosts (Figure 4). A similar change in MERS-CoV was not observed; however, MERS-CoV currently shows only limited human-to-human transmission, with most known transmission chains ending after 2 to 3 human-to-human transmission events as shown in the studies of Assiri et al.^32^ and Cotten et al.^21^ MERS-CoV might not have experienced sufficient number of human replication cycles or have undergone the same level of selection for human transmission that OC43, 229E, and SARS-CoV-2 have experienced. For both OC43 and 229E coronaviruses moving to humans, the broad location of the positive changes across the spike protein sequence suggested that positive charge may be promoting several functions including receptor binding, furin cleavage, cell fusion, as well as antigenic changes or less-specific changes to avoid or promote ionic interactions during transmission. Of course, it would be important to perform a similar analysis of spike change with the other important coronaviruses (e.g., NL63, HKU1, and SARS). Unfortunately, either no direct animal reservoir has been identified for these coronaviruses, or sufficient numbers of complete genome sequences are not available to perform the analysis.

There is likely a limit to the accumulation of positively charged residues in the SARS-CoV-2 spike protein. Functional constraints exist, there may also be penalties associated with non-specific binding due to excess positive charge, and there are certainly charge influences on protein folding and higher order protein interactions.^33^ Our prediction is that the SARS-CoV-2 protein will reach some upper limit of charge defined by these constraints. Indeed, we observe that the majority of Omicron lineages encode spike proteins with a charge of −1.26, after more than 6 months of evolution (Figures 2A and 2B). A small fraction of genomes with more positive charge or less positive charge have appeared, but the global tendency across all reported genomes from June to September 2022 is a modest decline in the positive charge (Figure 3B), which suggests the upper limit to charge has been reached.

Could these changes in spike charge have occurred by chance and not be a response to selective pressure? Of the 20 standard AAs, only 2 AAs have negatively charged side chains, 2 AAs have positively charged side chains, while the remaining 16 AAs are neutral at pH 7.4. Assuming equal probability of any AA change, there is an 18/20 chance of a negative AA being substituted by a neutral or positively charged AA, and the majority of change opportunities would result in a loss of negative charge. However, natural selection is more complex because the genetic code uses 3 adjacent nucleotides to encode an AA, there are multiple encoding possibilities for each AA, the codon redundancy is not identical for each AA, and the number of nucleotide changes required to produce any particular AA change can be 1, 2, or 3. This has resulted in an evolved protein stability in the genetic code,^34^ with AA changes that maintain rather than change physical properties (negative, positive, polar, non-polar, aromatic) more likely based on the codon array^35^ and the nucleotide changes required for an AA change. For example, the probability of a negative AA to negative AA change is 0.333, while the probabilities of change of a negative AA to a non-polar, aromatic, polar, or positive AA are 0.051, 0.044, 0.028, and 0.044, respectively, with changes away from a negatively charged AA nearly 10-fold less likely to occur than conserving the negative charge at that position.^35^ For these reasons, it appears that the accumulation of positive charge on spike protein has not occurred by chance and is likely providing some selective advantage for the virus. It should also be noted that the observed charge changes in exposed virion proteins seem to be limited to spike. Two additional SARS-CoV-2 proteins are externally exposed, the E protein (ORF4) and the M protein (ORF5), showing no consistent change in the charge of either of these proteins across the 2 years of the epidemic (results not shown).

We asked if the increased charge pattern we report for the Spike protein might extend to other proteins on the SARs-CoV-2 virion. There are 4 structural proteins in the SARS-CoV-2 virion, Spike (S), the orf4 envelope (E) protein, the orf5 membrane (M) protein, and the orf9 nucleoprotein (N), with only S, E, and M proteins exposed on the surface of the virion.^36^ We have monitored total protein charge for the 3 virion non-spike structural proteins (Figure S3). Unlike what was observed with the spike protein, there were patterns in the evolution of charge of the E, M, and N proteins, which varied. The virion-exposed E protein did not show changes in charge and has remained with a calculated charge (pH 7.4) of 1.2 (Figure S3A). The virion-exposed M protein showed a charge of 7.34 in the original B, Alpha, and Beta lineages, which dropped to charge of 6.34 in the Delta lineages and has the original charge of 7.34 in the Omicron lineages (Figure S3B). The virion non-exposed N protein showed an original charge of 23.35 in the B lineage, which increased slightly to 24.32 in the Delta and is currently at charge 25.35 in the Omicron lineages. Thus, the already highly positively charged RNA binding protein showed a small increase in positive charge (Figure S3C). Each of the viral structural proteins showed a different charge evolution pattern as described above, and thus, it appears that the steady increase in spike charge is a specific feature of the spike protein and not observed with all SARS-CoV-2 structural proteins.

Obermeyer et al. documented AA substitutions associated with SARS-CoV-2 fitness.^37^ Consistent with the idea that the increase in positive charges is not by chance, of the top 20 substitutions increasing SARS-CoV-2 fitness, 14 substitutions were in the spike protein, among which 4 were changes that increased positive charge while only 1 of 14 introduced a negative charge in spike.^37^

We have summarized the most frequently observed changes in the spike protein in Table S4, including the consequences of the AA changes (to positive, to negative, or neutral) and indicated changes in epitopes or to the receptor binding domain. One simple model is that the changes are in exposed residues that simply increase positive charge for improved transmission (e.g., to increase affinity to the ACE2 receptor). Mehra and Kepp have performed a detailed analysis of AA residue solvent exposure calculated from a set of spike protein structures.^38^ A useful metric used by Mehra and Kepp is the relative solvent accessible surface area (RSA) observed for that residues, with low values indicating structurally inaccessible (buried) AA residues and higher values indicating increasing tendency for surface exposure. A summary of the range of exposure displayed by positions that have been changed in spike is included in Table S4. For 33 AA changes with available RSA data, 18 changes alter charge (54.5%), and 12 of the 18 charges change toward a more positive charge (66.7%), indicating a modest trend toward positive changes. Of the 18 AA changes that alter charge, 11 of these show a range of RSA values from buried to exposed depending on the structure conditions, and only 2 AA changes involving charge are in consistently exposed positions (N440K, T478K). Thus, only a subset of these charge-changing positions are in consistently accessible regions of the protein. The variety of structural changes observed with these AA changes supports the idea that the spike charge changes are not limited to surface accessible regions of the spike protein and are consistent with the idea that spike protein charge may be involved in multiple processes (protein folding and stability, ACE2 binding, antibody avoidance, furin cleavage).

Natural selection could be acting on multiple features of the spike protein. The necessity to avoid host immune responses is likely to be the major selective force acting on the virus. This results in the AA changes, which in turn are determined by epitopes. The selection for increased charge in the spike protein is probably occurring in the background, not as a major shift needed to bypass immune responses. However, the increase in charge may improve survival and transmission in humans in subtle ways, and this advantage, when multiplied over the millions of infections, can provide some of the growth and infection advantages seen by new SARS-CoV-2 variants. It is proposed that the N764K, N856K, and N969K substitutions (all increasing spike positive charge) may enhance S1-S2 subunit interactions after proteolytic processing of the spike protein, resulting in reduced S1 shedding and improving transmission.^39^ Increased charge may also alter receptor interactions. In the Omicron (BA.1) spike protein, the Q493R and Q498R substitutions are predicted to allow two additional salt bridges with ACE2 receptor position 35Glu and 38Glu.^40^ Indeed, looking at the timing of charge shifts in each major lineage, the changes to a more positive charge accumulate later than the changes that first allow a lineage to emerge and dominate global infections. In this model, the primary spike changes are driven by immune selection and allow a new lineage to bypass existing immune responses. Once a successful new variant emerges, the large number of new infections allows selection for the accumulation of beneficial positive charge changes. The similar pattern of increased positivity of spike protein in other coronaviruses that have moved between animals and humans (OC43, 229E, Figure 4) suggests that the change in surface protein charge may be a more general phenomenon with coronaviruses and might be a useful parameter to examine when monitoring zoonosis. This study provides a framework to monitor viral evolution through changes in biochemical properties, which can be easily applied to other viruses important to public and global health. An important note is that our analyses on viral spike protein biochemical properties to monitor virus evolution are not meant to replace traditional phylogenetic analyses. The observed pattern of biochemical property changes should completement phylogenetic signals. However, in situations where there are limited sequences available to produce reliable phylogenetic signals (e.g., the 229E and OC43 viruses examined in Figure 4), this kind of analysis using virus biochemical properties from different host species would certainly help provide important information on the virus evolution, zoonosis, as well as aiding the prediction of patterns of viral changes.

In conclusion, our study provides a novel analytical framework to monitor viral evolution through changes in biochemical properties, which can be easily applied to other viruses important to public and global health. We also showed that natural virus evolution is more complicated and may involve multiple factors including immune selection, as well as spike protein biochemical properties. The observation of an increase of SARS-CoV-2 spike protein charge over time provides useful information for future vaccine and therapeutic development.

### Limitations of the study

Our analysis was limited to available data, and many of the SARS-CoV-2 genome sequences available have an incomplete spike coding region, which may introduce bias in our analysis. Our conclusions about zoonosis of non-SARS-CoV-2 coronaviruses are limited to available viruses with available sequence data, and for some of the coronavirus, no clear animal source has been identified, or insufficient sequence data are available to explore charge differences. Nonetheless, we believe the analyses reported here are a valid description of the spike charge changes.

## STAR★Methods

### Key resources table

**Table:** 

REAGENT or RESOURCE	SOURCE	IDENTIFIER
**Deposited data**		

Sequence data. See STAR Methods section	GISAID (cited in mansucript)	GISAID accession numbers can for the data used be downloaded from here: https://www.dropbox.com/s/b2k83ypsez2papj/Supplemental_Table_2_GISAID_accessions.csv.zip?dl=0
Sequence data	GenBank	GenBank accession numbers and details are described in Table S4.

**Software and algorithms**		

Code used to perform the analysis and generate figures is gathered in a single GitHub repository (see third box).	Written by co-authors	https://github.com/mlcotten13/SARS-CoV-2_spike_charge

### Resource availability

#### Lead contact

Further information should be directed to the lead contact, Matthew Cotten (matthew.cotten@lshtm.ac.uk).

#### Materials availability

This study did not generate new unique reagents.

### Method details

SARS-CoV-2 genomes sequences were obtained as a fasta file from GISAID^42^ with collection dates to 15 November 2022. Spaces in fasta IDs were removed using the command: sed (sed -i -e 's//_/g' msa_xxxx.fasta) and genomes were classified using Pangolin^43^ with the most recent database updates (pangolin v4.1.3, pangolin-data v1.16, constellations v0.1.10 and scorpio v0.3.17).

### Quantification and statistical analysis

The spike coding region from each genome, if present and intact (no Ns) was retrieved and translated into protein. Features of the spike protein that could be quantitated from the protein sequence were determined using the ProteinAnalysis functions from BioPython.^44^ These features included charge at pH 7.4 (and several other physiologically relevant pHs), Kyle and Doolittle GRAVY score^9^ (a measure of hydrophobicity), an instability index derived from dipeptide content,^10^ the total percent helix, fold or sheet properties of the protein and the total fractions of individual amino acids and fractions of di-amino acids. A matrix of all spike protein features plus collection date, and lineage was prepared and used for analysis. Similar analyses were performed for other coronaviruses such as 229E, OC43 and MERS-CoV by retrieving all complete genomes available from GenBank (15 June 2022). The spike protein was also extracted using the same method as aforementioned. Additional details are provided in the figure legends.

### Additional resources

No additional resources were used.

## Data Availability

• Data. A listing of the GenBank accession numbers for the data used in Figure 4 can be found in the Table S1. A listing of the 7,635,890 GISAID accessions that met the quality criteria and were used in the analysis can be found in the Table S2 which can be retrieved from the following link: https://www.dropbox.com/s/b2k83ypsez2papj/Supplemental_Table_2_GISAID_accessions.csv.zip?dl=0. • Code. The python scripts and Jupyter notebooks used to generate the figures can be found in the manuscript GitHub repository: https://github.com/mlcotten13/SARS-CoV-2_spike_charge. • All other items. No additional items are listed.

## Supporting citations

The following references appears in the Supplemental information: ^45^^,^^46^.
