# Supplementary material for: Evolution of increased positive charge on the SARS-CoV-2 spike protein may be adaptation to human transmission
Source: iScience. 2023 Feb 18;26(3):106230. doi: 10.1016/j.isci.2023.106230 (PMC9937996; doi:10.1016/j.isci.2023.106230)
Supplement: Document S1. Figures S1–S4 and Tables S3 and S4 [file mmc1.pdf]

iScience, Volume *26*

Supplemental information

Evolution of increased positive charge on the SARS-CoV-2 spike protein may be adaptation to human transmission

Matthew Cotten and My V.T. Phan

Supplemental Information

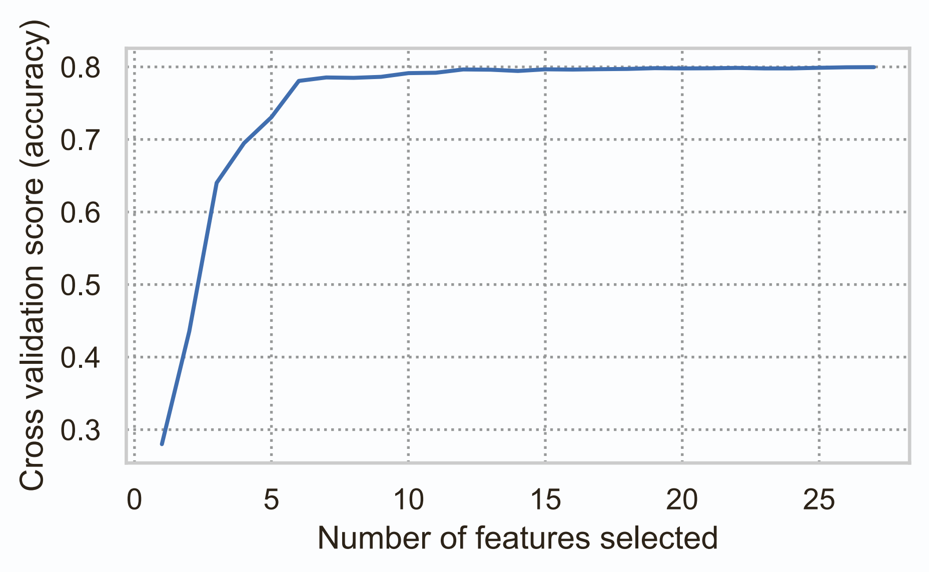

Supplemental Figure 1. Contribution of Spike protein features to lineage classification accuracy, related to Figure 1. For the spike protein feature matrix used to generate Figure 1A, lineage classification was measured as a function of the number of features used for classification. The combined accuracy of classification as a number of features used for the classification was determined using the SKLearn feature selection module RFECV. The plot shows cross validation scores (accuracy of lineage classification) as a function of the number of features selected.

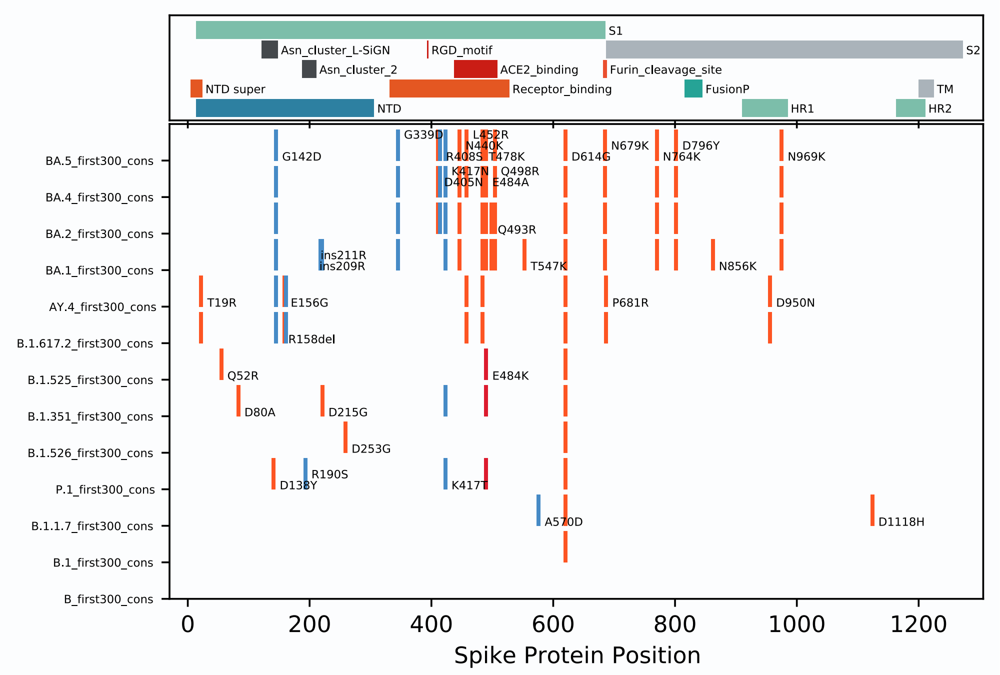

Supplemental Figure 2. Location of charged amino acid changes in the spike protein, related to Figure 2. The spike protein sequences encoded by the first 300 reported genomes for the indicated SARS-CoV-2 lineages were collected, and charged amino acid changes from the original B lineage spike sequence were plotted. Charge changes were colored from dark blue (change from positive to negative charged amino acid (AA)), blue change from neutral to negative charged AA), orange (change from neutral to positive charged AA) and red (change from negative to positive

charged AA). Substitutions are indicated by original AA/position in reference sequence spike/novel AA. The GenBank NC_045512 genome was used as reference. Key spike protein features of the SARS-CoV-2 spike protein are shown in the upper panel of the figure.

Changes in protein charge of structural proteins E, M and N.

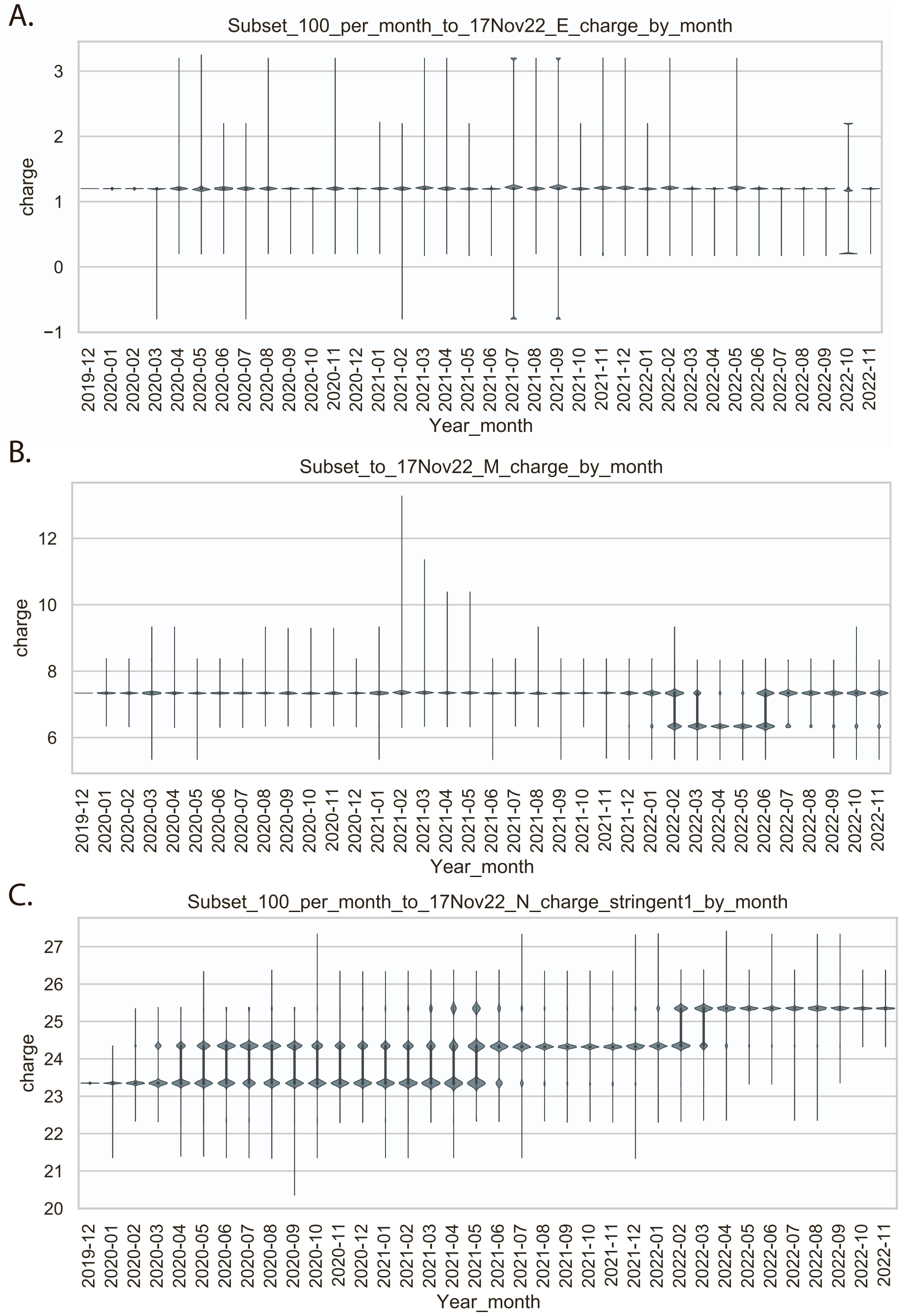

Supplemental Figure 3. Changes in SARS-CoV-2 structural proteins E, M and N across the 3 years of the epidemic, not related to any figure but added in response to a Reviewer's request. A thinned set of SARS-CoV-2 genome sequences was prepared by collecting the first 10 genomes deposited per country per month for all GISAID genomes available up to 15 November 2022. The E,

M and N coding sequences were extracted, and total protein charge at pH 7.4 was calculated. Violin plots of protein charge by month of epidemic are shown. Panel A: E protein charge, panel B: M protein charge, panel C: N protein charge.

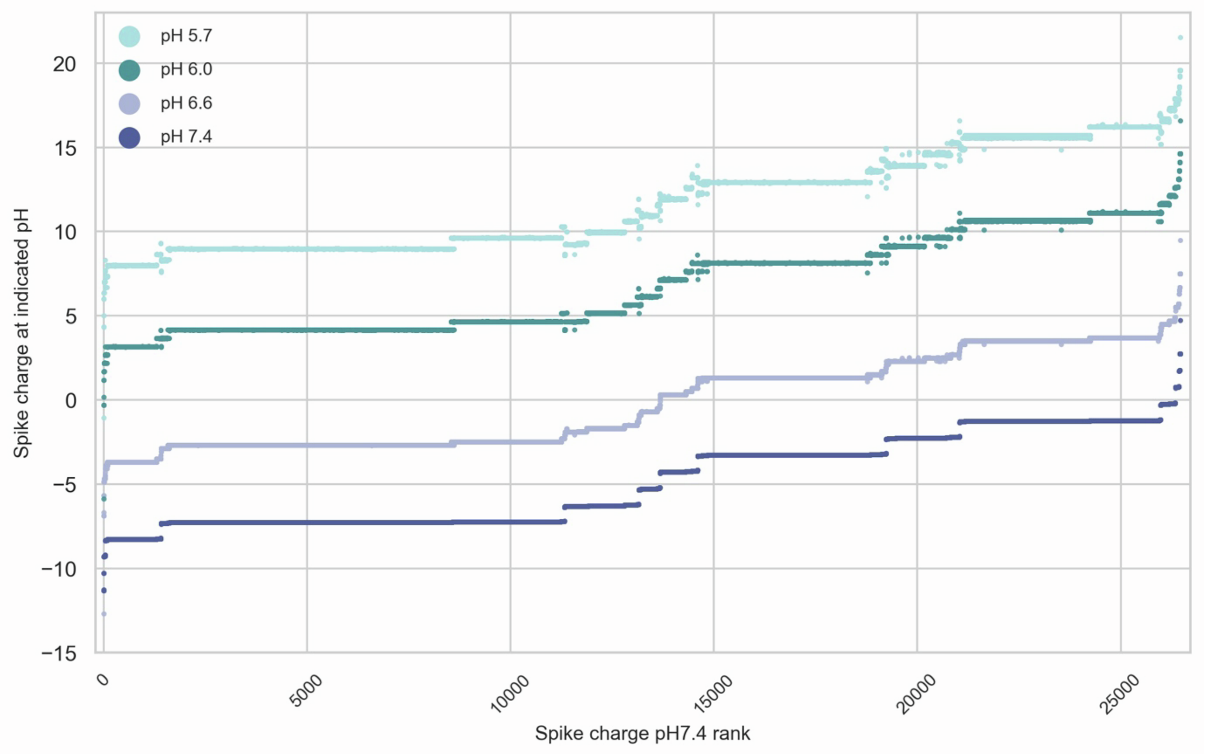

Supplemental Figure 4. Spike charge changes as function of pH, not related to any figure but added in response to a Reviewer's request. A thinned set of spike sequences was prepared from genomes sampled across the epidemic. From the entire set of genomes available on 17 Nov 2022, the first 10 genomes per month and per country were gathered. This provided a thinned set of genomes that represented all lineages across time. From this set of the genomes, the spike sequences, if intact were gathered (spike sequences in set: 26498) and the total spike charges across a range of pH values were calculated at the indicated pHs. The values for each pH were plotted by order of pH 7.4 charge value and values calculated at pH 6.6, 6.0 and 5.7 were plotted in the same rank order.

Supplemental Table 3. pH in relevant human sites, ot related to any figure but added in response to a Reviewer's request.

Location	pH
Sputum	7.25
Nasal	6.4 to 6.9
Endobronchia	5.7 to 6.6
Duodenum	6
Terminal ileum	7.4
Caecum	5.7
Rectum	6.7

References: [1], [2]

Supplemental Table 4. Summary of Spike amino acid changes and consequences, not related to any figure but added in response to a Reviewer's request.

Position	AA Change	Consequence[1]	Epitope[2]	Other_feature	Variant	Pango_Lineage	Exposure[3]
13	S13I	neutral					
18	L18F	neutral			Gamma	P.1	buried
19	T19R	to positive			Delta	B.1.617.2	
20	T20N	neutral			Gamma	P.1	buried to exposed
26	P26S	neutral			Gamma	P.1	exposed
67	A67V	neutral	yes		Omicron	B.1.1.529	
69	del69/70 (HV)	to negative	yes		Alpha	B.1.1.7	
80	D80A	to positive			Beta	B.1.351	buried to exposed
95	T95I	neutral			Omicron	B.1.1.529	
138	D138Y	to positive			Gamma	P.1	buried to exposed
142	G142D	to negative	yes		Delta Omicron	B.1.617.2 B.1.1.529	buried to exposed
152	W152C	neutral					
154	E154K	to positive					buried to exposed
190	R190S	to negative			Gamma	P.1	buried
212	L212I	neutral	yes		Omicron	B.1.1.529	
214	E insertion @214	to negative			Omicron	B.1.1.529	
214	P insertion @214	neutral			Omicron	B.1.1.529	
214	E insertion @214	to negative			Omicron	B.1.1.529	
215	D215G	to positive			Beta	B.1.351	buried to exposed
246	R246I	to negative					buried to exposed
339	G339D	to negative	yes	RBD	Omicron	B.1.1.529	
346	R346K	neutral	yes	RBD	Mu	B.1.621	
346	R346T	to negative	yes	RBD	Omicron	BQ.1.1	
371	S371L	neutral	yes	RBD	Omicron	B.1.1.529	
373	S373P	neutral	yes	RBD	Omicron	B.1.1.529	
375	S375F	neutral	yes	RBD	Omicron	B.1.1.529	
417	K417N	to negative	yes	RBD	Beta Omicron	B.1.351 B.1.1.529	buried
417	K417T	to negative	yes	RBD	Gamma	P.1	buried
439	N439K	to positive	yes	RBD			buried
440	N440K	to positive	yes	RBD	Omicron	B.1.1.529	exposed
445	V445P	neutral	yes	RBD	Omicron	XBB.1	
446	G446S	neutral	yes	RBD	Omicron	B.1.1.529	
446	G446V	neutral	yes	RBD			
447	S447N	neutral	yes	RBD		B.1.526	
452	L452R	to positive	yes	RBD	Delta	B.1.617.2	buried
452	L452M	neutral	yes	RBD			
453	Y453F			RBD			
455	L455F	neutral	yes	RBD			
477	S477N	neutral	yes	RBD	Omicron	B.1.1.529	exposed
477	S477G	neutral	yes	RBD			exposed
478	T478K	to positive	yes	RBD	Delta Omicron	B.1.617.2 B.1.1.529	exposed
484	E484K	to positive	yes	RBD	Beta Gamma	.351 P.1 B.1.525 B.1.5	buried to exposed
484	E484A	to positive		RBD	Omicron	B.1.1.529	
484	E484Q	to positive	yes	RBD	Delta	B.1.617.1	buried to exposed
485	G485R	to positive	yes	RBD			
486	F486L	neutral	yes	RBD			
486	F486V	neutral	yes	RBD	Omicron	BQ.1.1	
490	F490S	neutral	yes	RBD			
493	Q493R	to positive	yes	RBD	Omicron	B.1.1.529	
494	S494P	neutral	yes	RBD			buried
496	G496S	neutral	yes	RBD	Omicron	B.1.1.529	
498	Q498R	to positive	yes	RBD	Omicron	B.1.1.529	
501	N501Y	neutral	yes	RBD	Alpha Beta Gamma Omicron	B.1.1.7 P.1 B.1.1.529	buried
505	Y505H	to positive	yes	RBD	Omicron	B.1.1.529	
547	T547K	to positive	yes		Omicron	B.1.1.529	
570	A570D	to positive			Alpha	B.1.1.7	buried
614	D614G	to positive	yes	Conformation	Alpha Beta Gamma Omicron	B.1.1.7 P.1 B.1.1.529	buried to exposed
655	H655Y	to positive	yes		Gamma Omicron	P.1 B.1.1.529	buried to exposed
679	N679K	to positive	yes	Furin_cleavage_site	Omicron	B.1.1.529	
681	P681H	to positive	yes	Furin_cleavage_site	Alpha Omicron	B.1.1.7 B.1.1.529	
681	P681R	to positive		Furin_cleavage_site	Delta	B.1.617.2	
701	A701V	neutral			Beta	B.1.351	exposed
716	T716I	neutral			Alpha	B.1.1.7	buried
764	N764K	to positive			Omicron	B.1.1.529	
796	D796Y	to positive			Omicron	B.1.1.529	
856	N856K	to positive			Omicron	B.1.1.529	
950	D950N	to positive			Delta	B.1.617.2	
954	Q954H	to positive			Omicron	B.1.1.529	
969	N969K	to positive			Omicron	B.1.1.529	
982	S982A	neutral			Alpha	B.1.1.7	buried
1027	T1027I	neutral			Gamma	P.1	buried
1118	D1118H	to positive			Alpha	B.1.1.7	

Footnotes.

1. Consequence on spike charge of amino acid change. Changes that result in increased positive charge at pH 7.4 are marked in red.

2. Amino acids that have been reported to be a part of antibody epitopes. Summary of data from references [3], [4], [5], [6], [7].

3. Derived from Figure 8 of reference [8]. For the 33 positions analyzed, "buried" indicates the relative solvent accessible surface area per site (RSA, as defined by Mehra and Kepp, 2022) remained below 0.5 for this positions for all 10 structures,"buried to exposed" indicates RSA values for that position varied from <0.5 to > 0. 5 for the 10 structures, "exposed" indicates RSA values for that position were > 0. 5 for the 10 structures.

References.

1. Fischer, H., and Widdicombe, J.H. (2006). Mechanisms of Acid and Base Secretion by the Airway Epithelium. J. Membr. Biol. *211*, 139–150. 10.1007/s00232-006-0861-0.

2. Fallingborg, J. (1999). Intraluminal pH of the human gastrointestinal tract. Dan. Med. Bull. *46*, 183–196.

3. Greaney, A.J., Starr, T.N., and Bloom, J.D. (2022). An antibody-escape estimator for mutations to the SARS-CoV-2 receptor-binding domain. Virus Evol. *8*, veac021. 10.1093/ve/veac021.

4. Greaney, A.J., Loes, A.N., Crawford, K.H.D., Starr, T.N., Malone, K.D., Chu, H.Y., and Bloom, J.D. (2021). Comprehensive mapping of mutations in the SARS-CoV-2 receptor-binding domain that affect recognition by polyclonal human plasma antibodies. Cell Host Microbe *29*, 463-476.e6. 10.1016/j.chom.2021.02.003.

5. Greaney, A.J., Starr, T.N., Barnes, C.O., Weisblum, Y., Schmidt, F., Caskey, M., Gaebler, C., Cho, A., Agudelo, M., Finkin, S., et al. (2021). Mapping mutations to the SARS-CoV-2 RBD that escape binding by different classes of antibodies. Nat. Commun. *12*, 4196. 10.1038/s41467-021-24435-8.

6. Planas, D., Saunders, N., Maes, P., Guivel-Benhassine, F., Planchais, C., Buchrieser, J., Bolland, W.-H., Porrot, F., Staropoli, I., Lemoine, F., et al. (2022). Considerable escape of SARS-CoV-2 Omicron to antibody neutralization. Nature *602*, 671–675. 10.1038/s41586-021-04389-z.

7. Obermeyer, F., Jankowiak, M., Barkas, N., Schaffner, S.F., Pyle, J.D., Yurkovetskiy, L., Bosso, M., Park, D.J., Babadi, M., MacInnis, B.L., et al. (2022). Analysis of 6.4 million SARS-CoV-2 genomes identifies mutations associated with fitness. Science *376*, 1327–1332. 10.1126/science.abm1208.

8. Mehra, R., and Kepp, K.P. (2022). Structure and Mutations of SARS-CoV-2 Spike Protein: A Focused Overview. ACS Infect. Dis. *8*, 29–58. 10.1021/acsinfecdis.1c00433.
